# Dendritic Cells (DCs) as “Fire Accelerants” of Hantaviral Pathogenesis

**DOI:** 10.3390/v11090849

**Published:** 2019-09-13

**Authors:** Günther Schönrich, Martin J. Raftery

**Affiliations:** Institute of Virology, Charité—Universitätsmedizin Berlin, Corporate Member of Freie Universität Berlin, Humboldt-Universität zu Berlin and Berlin Institute of Health, 10117 Berlin, Germany

**Keywords:** dendritic cells, hantaviruses, virus-induced immunopathogenesis, antiviral immune responses

## Abstract

Hantaviruses are widespread zoonotic pathogens found around the globe. Depending on their geographical location, hantaviruses can cause two human syndromes, haemorrhagic fever with renal syndrome (HFRS) or hantavirus pulmonary syndrome (HPS). HPS and HFRS have many commonalities amongst which excessive activation of immune cells is a prominent feature. Hantaviruses replicate in endothelial cells (ECs), the major battlefield of hantavirus-induced pathogenesis, without causing cytopathic effects. This indicates that a misdirected response of human immune cells to hantaviruses is causing damage. As dendritic cells (DCs) orchestrate antiviral immune responses, they are in the focus of research analysing hantavirus-induced immunopathogenesis. In this review, we discuss the interplay between hantaviruses and DCs and the immunological consequences thereof.

## 1. Introduction

In 1973, Ralph Steinman and Zanvil Cohn published their discovery of a novel cell type, which they called dendritic cell [1]. In the following years, it became apparent that dendritic cells (DCs) play a central role in initiating antigen-specific immunity and tolerance [2]. In 2011, Ralph Steinman was awarded the Nobel Prize for Physiology or Medicine for his discovery [3]. DCs are part of the mononuclear phagocyte system (MPS) that also includes monocytes and macrophages. These cells were originally grouped together because monocytes were regarded as the precursor cells of macrophages and DCs [4]. This perception has become more nuanced over time, and we now know that whereas monocytes can develop into cell types with similarities to macrophages and DCs, most macrophages undergo self-renewal whereas classical DCs are continuously generated from precursor cells that are distinct from monocytes [4].

Viral hemorrhagic fever (VHF) denotes a group of zoonotic human diseases that are caused by different RNA viruses including hantaviruses [5,6]. Hantaviruses belong to the *Hantaviridae*, a virus family within the order *Bunyavirales* [7]. They circulate worldwide in different natural hosts depending on the geographical region [8]. Humans are usually infected with pathogenic hantaviruses after inhalation of aerosols derived from excreta of infected rodents [9,10]. Person-to-person transmission has only been described for Andes virus (ANDV) [11,12,13,14], the main etiologic agent of HPS in South America [15]. Hantaviruses found in the New World/the Americas such as Sin Nombre virus (SNV) and ANDV cause hantavirus pulmonary syndrome (HPS) with high case fatality rates up to 40% [16]. In contrast, hantaviruses in Europe (e.g., Puumala virus, PUUV) and in Asia (e.g., Hantaan virus, HTNV) are associated with hemorrhagic fever with renal syndrome (HFRS), a form of VHF with generally lower fatality rates [17]. So far, neither specific therapy nor vaccines approved by the Food and Drug Administration (FDA) are available [18].

Pathogenic hantaviruses elicit an immune response in humans that not only provides antiviral defence mechanisms, but also contributes to virus-induced immunopathogenesis and possibly viral dissemination [19,20]. In fact, hantaviruses, like other VHF viruses, target DCs and other cells of the MPS for replication, dissemination and shielding from immune attack [21]. Here, we review the role of DCs in hantaviral immunopathogenesis.

## 2. DC Subsets

DCs are heterogeneous and divided into two main groups: plasmacytoid DCs (pDCs) and classical DCs (cDCs), which are also often called conventional DCs or myeloid DCs. In contrast to cDCs, pDCs are of lymphoid origin. They circulate in the blood and are detected in lymphoid tissue. pDCs act as “natural interferon-producing cells” during viral infections. They have a unique capacity for rapidly releasing interferon (IFN) type I in response to microbial stimuli, thereby facilitating virus clearance [22]. However, pDCs are resistant to most viral pathogens [23].

cDCs reside either in lymphoid organs or in barrier tissue such as skin (e.g., dermal and interstitial DCs) and mucosa of lung and intestine. In the peripheral blood of healthy adults, DCs represent only a very small subset (0.5%–1.5% of mononuclear cells) [24]. The cDCs in the peripheral tissue are migratory, taking up antigen in the periphery by phagocytosis and migrating via afferent lymph to the draining lymph nodes. In fact, DCs are equipped with numerous different pattern recognition receptors (PRRs) that act as sensors of pathogen-associated molecular patterns (PAMPs) [25]. Upon receiving signals via PRRs, migratory cDCs acquire a mature phenotype with high levels of major histocompatibility complex (MHC) class II molecules and co-stimulatory molecules on the cell surface [26]. After migration to the draining lymph nodes, mature cDCs present antigen to CD4+ and CD8+ T lymphocytes, thereby initiating powerful adaptive immune responses. Importantly, certain DC subsets are able to present exogenous antigens to CD8+ T cells, a process called cross-presentation [27,28]. In this way, cDCs can bypass viral evasion of MHC class I presentation. At sites of inflammation, an additional subset of DCs called inflammatory DCs is observed [29]. Inflammatory DCs are derived from monocytes that are recruited into inflamed tissue and represent the most abundant DC population during inflammation. Monocyte-derived DCs that have been generated in vitro are routinely used as a model system to analyse virus-infected myeloid DCs [30]. This approach revealed an astonishingly broad range of different viruses infecting monocyte-derived DCs, setting them apart from pDCs [31].

## 3. Hantavirus Infection of DCs

SNV was discovered during an outbreak in the Four Corners states (New Mexico, Colorado, Utah and Arizona) of southwestern United States [32]. Subsequent immunohistochemical testing of tissues from patients that died of SNF infection revealed hantaviral antigen in follicular dendritic cells (FDCs) and macrophages [33]. FDCs are localized in follicles of the B-cell zone of lymphoid tissue [26] but are not related to leucocyte DCs, although they can retain antigen and virions for a long time [34].The permissivity of DCs for Old World hantaviruses was first demonstrated in vitro by infecting DCs from various sources (monocyte-derived DCs, DCs derived from CD34+ progenitor cells) with HTNV [35]. The monocyte-derived human DCs are generated by culturing CD14+ monocytes, isolated from peripheral blood mononuclear cells (PBMC) in the presence of granulocyte-macrophage colony-stimulating factor (GM-CSF) and interleukin-4 (IL-4) [36]. cDCs isolated from the peripheral blood of healthy donors are also susceptible to Old World hantaviruses [35,37]. Other researchers demonstrated that a clinical isolate of ANDV infects human monocyte-derived DCs in vitro [38]. Not only monocyte-derived DCs, but also monocytes themselves and macrophages are susceptible to infection with Old World and New World hantaviruses [19,37,39,40]. Hantavirus tropism for cells of the MPS is not restricted to humans. Rat bone marrow-derived DCs and macrophages are also permissive for Seoul virus (SEOV), an Old World hantavirus [41]. These findings indicate that hantaviruses target DCs not only in humans but also in their natural hosts.

## 4. DCs as a Trojan Horse for Hantaviruses

The hallmarks of VHF, dysfunction and leakage of the endothelial barrier and bleeding, are associated with infection of endothelial cells (ECs) [42,43]. ECs also play a fundamental role in hantavirus-induced pathogenesis [44,45]. In fact, the hantavirus-infected ECs are a hot spot for immunopathological mechanisms such as formation of neutrophil extracellular traps (NETs) [46,47,48]. Hantavirus infection has been shown to strikingly upregulate the number of circulating endothelial progenitor cells (cEPCs) in the blood [49], probably as a consequence of damage repair rather than mobilization of infected cells [50]. At the moment, it is unclear by which means pathogenic hantaviruses travel from the lung to the endothelial cell barrier due to the lack of appropriate animal models [51].

Dissecting hantaviral dissemination, however, is key for understanding hantavirus-induced disease. The capacity to disseminate in the body differentiates pathogenic hantaviruses from their apathogenic cousins. It is possible that initial infection is via uptake of infectious particulate matter by migratory cDCs in the lungs, resulting in activation, migration and finally dissemination. In accordance, lung cDCs are in close contact with the respiratory epithelium and the alveolar interstitium and “snorkel” through the epithelial tight junctions, with outward facing of their dendritic projections into the airway lumen [52]. In this way, lung cDCs may be highjacked by hantaviruses immediately after inhalation of contaminated aerosols. 

It is unlikely that free-floating virions in the serum are sufficient to infect ECs in the microvasculature. Hantaviruses have been shown to adhere to platelets [53] and probably many other cell types due to their binding to common integrins [54]. However, these interactions are mostly passive, as the cells are not permissive to infection. The virus is thus a passenger on only a few of a vast horde of cells. These free or loosely attached virions will be vulnerable to rapid inactivation by innate immune mechanisms. However, viruses that productively infect and activate mobile cells of the MPS such as monocytes or cDCs are both protected from the nascent immune response and amplified by the replication in this cell type. Moreover, although hantavirus infection of DCs does not cause cell lysis or any other obvious cytopathic effect, it does cause maturation [35] and enhanced migration [37]. Thus, the circulating hantavirus-infected MPS cells may act as Trojan Horse that allows the pathogen to hide from immune attack and travel to EC monolayers. Innate signalling through PRRs may prompt hantavirus-infected cDCs and monocytes to leave the circulation, and during the process of migration and diapedesis through the endothelial barrier, thus pass infection on to ECs. 

## 5. Immunological Consequences of Hantavirus Infection of DCs

The importance of hantavirus-induced immune responses for hantaviral pathogenesis is strongly suggested by the onset of symptoms, which usually occurs after the peak of virus replication and often after the virus has ceased to replicate in most compartments. In line with this view, the magnitude of the pulmonary cytotoxic lymphocyte response correlates with disease severity in HFRS patients [55]. Similarly, in HPS patients, the strength of the hantavirus-specific T cell responses mirrors disease severity [56]. Antigen presentation is the cornerstone of adaptive antiviral defence and immunological memory. In particular, cDCs are extremely potent T-cell stimulators. Accordingly, they are called professional antigen-presenting cells, whereas most other cell types, such as ECs are amateurs regarding antigen presentation [57]. Although ECs can express low levels of MHC class II molecules, they are not capable of effective co-stimulation and do not produce the relevant costimulatory cytokines such as IL-12 [58]. Upon infection with hantavirus and other viruses, however, ECs express high levels of inhibitory molecules such as PD-L1, which functions as a ligand for the programmed cell death protein (PD-1, also referred to as CD279) on T cells [59,60]. This most likely reflects an attempt to prevent cytotoxic attack on the endothelial barrier and its deleterious consequences.

In contrast, hantavirus infection of monocyte-derived DCs in vitro results in activation and maturation into highly effective professional antigen-presenting cells [35]. Pathogenic hantaviruses typically upregulate classical MHC class I and class II molecules on monocyte-derived DCs, with consequent improvement of presentation to CD8+ and CD4+ T cells, respectively. Moreover, upon infection with hantavirus, monocytes and monocyte-derived DCs upregulate chemokine receptor CCR7, which is important for migration from the lymphatics to the draining lymph nodes, where T cells are stimulated [37]. After hantavirus infection in vitro, monocyte-derived DCs acquire the capacity to cross-present antigen to CD8+ T cells [61]. Cross-presentation plays a pivotal role the induction of effector cytotoxic antiviral CD8+ T cells [27]. Recently, cross-presentation has also been demonstrated for monocyte-derived DCs found in tumour ascites from cancer patients [62]. Thus, monocytes recruited to lung tissue during the early phase of hantavirus infection could take up hantaviral antigen, develop into inflammatory DCs and cross-prime a strong antiviral cytotoxic T cell response after migration to the draining lymph nodes.

Hantavirus-infected DCs upregulate CD80 and CD86, which both bind to the costimulatory receptor CD28 on T cells. Besides costimulatory ligands, monocyte-derived DCs infected with hantavirus also strongly upregulate PD-L1 and PD-L2, the two known PD-1 ligands [59]. In accordance, increased amounts of both soluble costimulatory molecules (sCD86) and soluble inhibitory molecules (sPD-L1, sPD-L2) are found in sera from hantavirus-infected patients [59]. Longitudinal analysis of T cells during acute hantavirus infection, however, revealed no PD-1 expression. Moreover, it has been demonstrated that CD80 interacts with PD-L1 on the surface of DCs in cis [63] and prevents PD-L1 from binding to PD-1 in trans [64,65]. These findings suggest that hantaviral induction of PD-L1 and PD-L2 expression is ineffective as a T cell brake [66]. In accordance, hantavirus-induced activation of bystander CD8+ T cells is observed after hantavirus infection of PBMCs in vitro and requires CD14+ cells [59]. Although CD14 is considered as a marker for monocytes and tissue macrophages, it is also found on human inflammatory DCs [67]. Thus, inflammatory DCs may drive bystander activation of CD8+ T lymphocytes during acute virus infection. These cells are activated independently from the T cell receptor (TCR) and show innate-like cytolytic activity that can cause tissue injury [68] or secrete proinflammatory cytokines such as IFN-γ [69]. Moreover, hantavirus-induced bystander activation affects also B cells, as recently described for HPS patients [70]. Similar to T cells and B cells, natural killer (NK) cells can rapidly respond to hantavirus infection beyond the level that is normally observed with other virus infections [20,71]. In turn, these bystander-activated immune cells may license uninfected DCs cross-presenting hantaviral antigen to initiate hantavirus-specific (TCR-dependent) T-cell activation and expansion [72,73].

**Figure 1:**
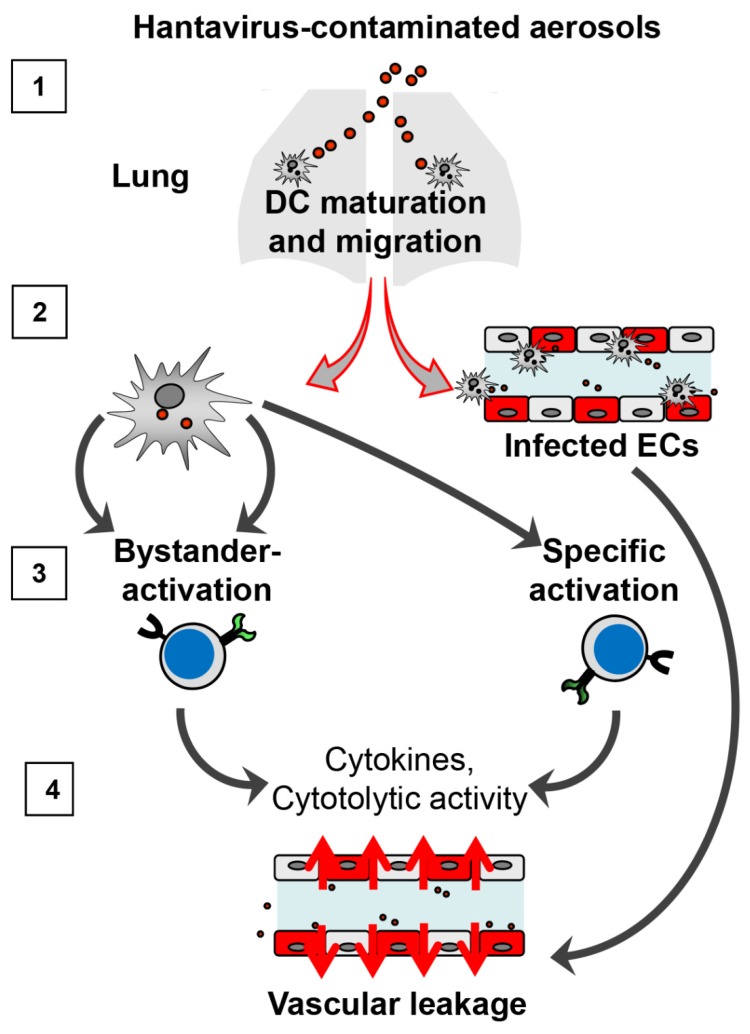
Dendritic cells (DCs) as “fire accelerants” in hantavirus-induced pathogenesis. (1) After inhalation, pathogenic hantaviruses may productively infect DC subsets in close contact with the respiratory epithelium/alveolar interstitium, thereby inducing DC maturation and migration. (2) Hantavirus-infected DCs can act as shuttles that safely transport virions to endothelial cells (ECs), the main arena of hantavirus-induced pathogenesis, resulting in vascular leakage, and lymphoid tissue, where immune cells are activated. (3) Hantaviruses-infected DCs cause massive bystander activation of immune cells (CD8+ T cells, B cells, NK cells). Bystander-activated immune cells may in turn license DCs cross-presenting hantaviral antigen to initiate powerful hantavirus-specific adaptive immune responses. (4) Both bystander-activated cells and hantavirus-specific immune cells may further aggravate vascular damage by attacking ECs not protected by expression of inhibitory molecules such as PD-L1 or by secreting cytokines that enhance vascular permeability (e.g., IFN-γ).

Taken together, hantavirus-infected DCs act as “fire accelerants” in hantaviral pathogenesis in several aspects (Figure 1). Firstly, DCs may play a pivotal role in initial uptake and replication of pathogenic hantavirus. Because of this early encounter, DCs mature and start to migrate. Secondly, these migratory hantavirus-infected DCs can act as shuttles that safely transport infection to ECs, the main arena of hantavirus-induced vascular leakage. Thirdly, hantaviruses-infected DC subsets can cause massive bystander activation of immune cells (CD8+ T cells, B cells, NK cells). Bystander-activated immune cells could then themselves license DCs that cross-present hantaviral antigen and induce powerful hantavirus-specific immune responses. Finally, bystander-activated and hantavirus-specific immune cells may both contribute to vascular damage by attacking ECs not protected by expression of inhibitory molecules such as PD-L1 or by secreting cytokines that enhance vascular permeability (e.g., IFN-γ).

## 6. Conclusions

DCs are major players in hantavirus-induced immunopathogenesis. Their infection results in immunological responses that are disproportionate to the level of infection. Whether infection of other cell subsets in the peripheral blood also contributes to pathology is unclear, or even if other cells are capable of supporting viral replication. The detailed molecular mechanisms that induce hantavirus-induced bystander activation of immune cells and the pathology resulting thereof also has to be clarified. Based on this research, strategies could be developed that prevent misdirected immune responses induced by pathogenic hantaviruses.
